# Zearalenone in the Intestinal Tissues of Immature Gilts Exposed *per os* to Mycotoxins

**DOI:** 10.3390/toxins7083210

**Published:** 2015-08-18

**Authors:** Łukasz Zielonka, Agnieszka Waśkiewicz, Monika Beszterda, Marian Kostecki, Michał Dąbrowski, Kazimierz Obremski, Piotr Goliński, Maciej Gajęcki

**Affiliations:** 1 Department of Veterinary Prevention and Feed Hygiene, Faculty of Veterinary Medicine, University of Warmia and Mazury in Olsztyn, Olsztyn 10-719, Poland; E-Mails: michal.dabrowski@uwm.edu.pl (M.D.); obremski@uwm.edu.pl (K.O.); gajecki@uwm.edu.pl (M.G.); 2 Department of Chemistry, Poznań University of Life Sciences, Wojska Polskiego 75, Poznań 60-625, Poland; E-Mails: agat@up.poznan.pl (A.W.); monika.beszterda@up.poznan.pl (M.B.); marian.kostecki@up.poznan.pl (M.K.); piotrg@up.poznan.pl (P.G.)

**Keywords:** zearalenone, *per os*, metabolites, intestine, carryover, gilts

## Abstract

Zearalenone and its metabolites, α-zearalenol and β-zearalenol, demonstrate estradiol-like activity and disrupt physiological functions in animals. This article evaluates the carryover of zearalenone and its selected metabolites from the digesta to intestinal walls (along the entire intestines) in pre-pubertal gilts exposed to low doses of zearalenone over long periods of time. The term “carryover” describes the transfer of mycotoxins from feed to edible tissues, and it was used to assess the risk of mycotoxin exposure for consumers. The experimental gilts with body weight of up to 25 kg were *per os* administered zearalenone at a daily dose of 40 μg/kg BW (Group E, *n* = 18) or placebo (Group C, *n* = 21) over a period of 42 days. In the first weeks of exposure, the highest values of the carryover factor were noted in the duodenum and the jejunum. In animals receiving pure zearalenone, the presence of metabolites was not determined in intestinal tissues. In the last three weeks of the experiment, very high values of the carryover factor were observed in the duodenum and the descending colon. The results of the study indicate that in animals exposed to subclinical doses of zearalenone, the carryover factor could be determined by the distribution and expression of estrogen receptor beta.

## 1. Introduction

Mycotoxicological research focuses on compounds that exert adverse effects on human health [1] and that cause significant losses in livestock and companion animals. Zearalenone is one of the most interesting mycotoxins. This nonsteroidal estrogenic mycotoxin and mycosteroid is produced by fungi of the genus *Fusarium* [2]. In the literature, it is also referred to as F-2 toxin, fermentation estrogenic substance and *trans*-zearalenone. Zearalenone is a specific hormone controlling sexual reproduction in *Fusarium* spp. (sexual stage *Gibberella zeae*). Under optimal moisture and temperature conditions, ZEN is produced by various *Fusarium* species, including *Fusarium graminearum* (*Gibberella zeae*), *F. culmorum*, *F. equiseti*, *F. gibbosum*, *F. lateritium*, *F. avenaceum*, *F. moniliforme*, *F. tricinctum* and *F. roseum* [3].

The first reports about the pathogenic qualities of ZEN date back to the early 20th century [4]. Clinical symptoms of vulvovaginitis were observed in pigs fed moldy corn. In successive studies, a specific uterotrophic substance was isolated from the filaments of *Gibberella zeae* (*Fusarium graminearum*). In the 1960s, the same compound was isolated from corn inoculated with *Fusarium* spores, and it was named F-2 toxin. Spectrophotometry was used to determine the molecular structure of ZEN and to perform the first chemical synthesis of the compound [5]. Biological material contains ZEN, as well as its metabolites, including α-ZEL and β-ZEL. The estrogenic activity of α-ZEL is three-fold or even four-fold higher in comparison to ZEN.

The interactions observed between ZEN, its metabolites and major cell and/or tissue structures responsible for the maintenance of hormonal homeostasis (at the systemic and local level) indicate that ZEN belongs to the group of endocrine disrupting chemicals (EDCs) [6]. Zearalenone demonstrates both structural (the presence of a phenol ring) and functional similarity to endogenous estrogens [7]. Its hormonal properties are manifested by ZEN’s ability to interact with ERα and ERβ [8] and to modulate the expression and/or activity of enzymes participating in the synthesis of sex steroid hormones (e.g., P450scc, cholesterol side-chain cleaving enzyme; 3β-HSD, 3β-hydroxysteroid dehydrogenase; P450arom, aromatase) [9]. Zearalenone is an agonist of ERs when the concentrations of endogenous estrogens are low [10]. In pigs, biotransformation of ZEN is mostly a bioactivation process, which leads to the production of α-ZEL, a metabolite whose estrogenic activity is three- to four-fold higher in comparison to the parent compound. At high concentrations, ZEN competes with endogenous steroid hormones for binding sites on receptors and/or enzymes (e.g., PXR, pregnane X receptor; 3α-HSD, β-HSD), which participate in the chemical transformation of endogenous (including steroids) and exogenous compounds (including ZEN) [9]. Pre-pubertal gilts are most susceptible to ZEN’s endocrine-disrupting activity [9], which is determined by: (i) ZEN concentrations (during exposure to ZEN or ZEN intoxication), which can be 100- to 10,000-fold higher than the physiological levels of endogenous estrogens; (ii) expression of mRNA and ER proteins in the target cells of the reproductive system; and (iii) the functional immaturity of the feedback loop responsible for hormonal balance. The range and intensity of clinical symptoms, histological and anatomopathological changes that accompany ZEN-induced mycotoxicosis in pigs are determined by: (i) the animal’s sex, age, sexual maturity/phase of the reproductive cycle and physiological status; and (ii) ZEN concentrations in feed, period of exposure, type of exposure (single, chronic) and the presence of other mycotoxins or EDCs [6].

In the early stages of exposure to ZEN, histological and/or anatomopathological changes are observed mainly in the gastrointestinal tract and in estrogen-responsive tissues. Macroscopic changes in the volume and/or weight of the ovaries [8] and the uterus [11] were observed in pre-pubertal gilts and female dogs, even at NOAEL doses. Morphometric changes in the digestive tract were rarely reported under exposure of NOAEL doses of ZEN [12].

The latest research suggests that ZEN can contribute to: (i) histological changes in gastrointestinal walls [13]; (ii) changes in the activity of goblet cells and mucus production; (iii) changes in the activity of the local immune system [14]; (iv) changes in the metabolic profile [15]; and (v) changes in the rate of weight gain [16]. The above changes can be attributed to the carryover of ZEN from the gastrointestinal tract to selected tissues. The results of this study can be used to determine the probability and course of exposure to the analyzed mycotoxin (undesirable substance) [17].

The objective of this study was to evaluate the carryover of ZEN and its selected metabolites from the digesta to intestinal walls (along the entire intestines) in pre-pubertal gilts exposed to low doses of ZEN over long periods of time.

## 2. Results

Mycotoxin concentrations in the analyzed feed were below the sensitivity of the method (VBS). Similar observations were made in analyses of ZEN metabolites in the digestive tract and the liver. The concentrations of α-ZEL and β-ZEL were also below the sensitivity of the method. The concentrations of ZEN, α-ZEL and β-ZEL in the tissues of animals from the control group were also below the sensitivity of the method.

Throughout the experiment, the average daily gains of gilts reached 602 g (2.61 kg feed per kg of BW gain) in Group E and 576 g (2.72 kg feed per kg of BW gain) in Group C. No significant differences in average daily gains were found between groups.

The results of statistical analyses of ZEN concentrations in tissues in different intestinal segments and in the liver in successive weeks of exposure are presented in Table 1. Statistically-significant differences (*p* ≤ 0.05) in ZEN levels were observed only in the liver between Weeks I and IV (higher) and between Weeks II and V (higher). Highly significant differences (*p* ≤ 0.01) in ZEN concentrations were not noted only in the ascending colon. Significant differences were observed in the remaining tissues. In the duodenum, ZEN concentrations differed significantly between Week V (higher) and Weeks I to IV. In the jejunum, fluctuations in ZEN levels were observed between Week IV (higher) and the remaining weeks and between Week VI and Week IV (higher). Ileal levels of ZEN differed between Week VI (higher) and the remaining weeks of the experiment. In the cecum, significant differences in ZEN concentrations were observed between Week V (higher) and Weeks I to IV and between Week I (lower) and Weeks IV and VI. In the descending colon, ZEN levels differed between Weeks I and V (higher) and between Week VI (higher) and Weeks I and II.

**Table 1:** ZEN concentrations (ng/g) and CF values in tissues in different intestinal segments and in the livers of pre-pubertal gilts in successive weeks of exposure. Daily feed intake in a restricted feeding regimen (kg/day). Mean dietary ZEN concentrations per kg of feed (µg/kg feed).

Weeks of Exposure	Feed Intake (kg/day)	ZEN Intake (µg/kg feed)	Total ZEN Dose (µg/kg BW)	ZEN Concentrations ($\bar{x}$ and SD)/Carryover Factor (CF)							
				Duodenum	Jejunum	Ileum	Cecum	Ascending Colon	Transverse Colon	Descending Colon	Liver
**I**	1,1	1014	280	8.60 ** ± 1.58	9.87 ^●●^ ± 0.81	4.63 ^▲▲^ ± 1.20	4.54 **^,▲▲^ ± 2.09	1.53 ± 1.95	1.39 ^●●,▲▲^ ± 1.32	4.70 ^●●,^** ± 3.98	6.31 ^●,^**^,▲▲^ ± 6.16
				0.031	0.035	0.016	0.0162	0.0054	0.0049	0.0167	0.0225
**II**	1,3	972	560	12.34 ** ± 1.75	19.72 ^●●,▲▲^ ± 2.75	4.99 ^▲▲^ ± 1.64	4.25 **^,▲▲^ ± 0.32	1.59 ± 2.76	4.63 ± 3.86	6.80 ^●●^ ** ± 1.40	7.80 *^,▲▲^ ± 2.82
				0.022	0.035	0.009	0.0075	0.0028	0.0082	0.0121	0.0139
**III**	1,4	1014	840	12.07 ** ± 3.48	13.85 ^●●^ ± 0.48	7.22 ^▲▲^ ± 0.30	4.30 **^,▲▲^ ± 1.80	3.16 ± 1.79	3.94 ± 0.57	7.60 ^●●,^** ± 2.33	13.00 ± 1.78
				0.014	0.016	0.008	0.0057	0.0037	0.0046	0.0090	0.0154
**IV**	1,5	987	1120	21.68 ** ± 4.01	43.82 ± 7.63	4.44 ^▲▲^ ± 1.52	3.45 **^,▲▲^ ± 0.67	4.69 ± 0.45	7.37 ± 1.66	205.01 ± 94.70	18.20 ± 5.92
				0.019	0.039	0.004	0.0030	0.0042	0.0065	0.1830	0.0162
**V**	1,8	995	1400	128.18 ± 64.54	13.66 ^●●^ ± 3.73	3.37 ^▲▲^ ± 0.35	18.17 ± 5.02	3.40 ± 0.54	6.49 ± 0.96	177.03 ± 50.64	20.01 ± 2.43
				0.091	0.010	0.002	0.0129	0.0024	0.0046	0.1264	0.0142
**VI**	2,1	957	1680	80.74 ± 4.34	8.33 ^●●^ ± 1.12	27.52 ± 10.28	13.70 ± 1.17	2.46 ± 1.92	6.70 ± 0.25	112.01 ± 13.69	23.00 ± 0.79
				0.048	0.005	0.016	0.0081	0.0014	0.0039	0.0666	0.0136
**Average values**			ZEN	43.93	18.208	8.705	0.068	2.805	5.0086	85.525	14.72
			CF	0.0375	0.0233	0.0091	0.0089	0.0033	0.0054	0.0688	0.0159

Key: * and **: relative to Week V, ^●●^: relative to Week IV; **^▲▲^**: relative to Week VI; ^●^: at *p* ≤ 0.05; ^●●^, **, **^▲▲^**: at *p* ≤ 0.01.

The results of chromatographic analyses of digestive tract tissues sampled from pre-pubertal gilts exposed to ZEN revealed significant variations in mycotoxin levels between the examined tissues, weeks of exposure and CF values (Table 1). In the duodenum, the first section of the small intestine, ZEN concentrations were determined between 8.6 ng/g (Week I, CF = 0.031) and 128.2 ng/g (Week V, CF = 0.091), with a significant increase in Week VI relative to the remaining weeks of the experiment. In the jejunum, ZEN levels ranged from 8.3 ng/g (Week VI, CF = 0.005) to 43.8 ng/g (Week IV, CF = 0.039). The average ileal concentrations of ZEN between Week I and Week V were determined at 4.9 ng/g (CF ≈ 0.043), with a significant increase in Week VI (27.5 ng/g, CF = 0.016). In the cecum, the first segment of the large bowel, mycotoxin levels increased steadily to reach 18.17 ng/g in Week V (CF = 0.0129).

In the analyzed sections of the large intestine and the liver of experimental animals, ZEN levels in the descending colon increased significantly in Week IV, after which a drop in ZEN concentrations and CF values was observed (Table 1). The highest levels of ZEN were observed in the ascending colon at 4.7 ng/g (Week IV, CF = 0.0042), transverse colon at 7.4 ng/g (Week IV, CF = 0.0065), descending colon at 205 ng/g (Week IV, CF = 0.1830) and the liver at 23 ng/g (Week VI, CF = 0.0136). In the large intestine, ZEN concentrations increased from Week IV. The highest ZEN levels were noted in the duodenum at 128 ng/g (Week V, CF = 0.091) and in the descending colon at 205 ng/g (Week IV, CF = 0.1830).

## 3. Discussion

The amount of ZEN transported to the digestive tract of gilts exposed to low monotonic doses of ZEN has not been studied to date. Initial estimates revealed significant differences in comparison to tissues that had been exposed to higher mycotoxin doses. Low-dose mycotoxicoses do not produce subclinical symptoms of disease, but they may induce weakly-expressed changes (stimulation or a compensatory effect) [16] at the tissue or cell level. The observed variations and the associated uncertainty encourage new research to improve safety assessments and risk-informed decision making [18]. The carryover of ZEN to the gastrointestinal walls in gilts exposed to low doses of the mycotoxin over long periods of time was compared to the results of other studies performed on gilts.

Mycotoxins are absorbed [19], metabolized [20,21] and distributed mainly via the digestive system [22], which is particularly exposed to the toxic effects of those undesirable substances, including ZEN and its metabolites [23]. The majority of Fusarium mycotoxins are absorbed in the proximal section of the small intestine [24]. Those variations can be attributed to significant physiological differences across intestinal segments. For example, the lowest concentrations of mucus glycoproteins are found in the duodenum and the jejunum, which enhances the absorption of digesta across the intestinal wall [19] and makes it more available. Most carbohydrates are also absorbed in the proximal part of the small intestine [25], which facilitates the accumulation and, probably, biotransformation of mycotoxins in enterocytes [2]. The data regarding those processes and the involvement of various intestinal sections are highly diverse and incomplete. *In vitro* studies revealed that 51% [26] to 55% [12] of ingested ZEN is absorbed in the small intestine. The involvement of different intestinal segments in this process has not been described.

In this experiment, the highest amounts (%) of ZEN in the small intestine were observed in the first weeks of exposure (I to III). In the remaining weeks, ZEN was also accumulated in the duodenum and the descending colon. The carryover of ZEN to various intestinal segments differs from the transport of deoxynivalenol (DON), whose accumulation increases proportionally with time of exposure [22].

A study of ZEN and α-ZEL resorption demonstrated that the mycotoxin and its metabolite are absorbed in 65% from the duodenum [27] when animal feed is naturally contaminated with those compounds. It has been suggested that ZEN is reduced to α-ZEL and β-ZEL in the duodenal mucosa where β-ZEL is the predominant fraction [28]. The above results indicate that the duodenum is an important site for ZEN absorption, but not biotransformation. The latter seems to be confirmed by our study where ZEN metabolites were not determined in the duodenum. Unfortunately, all concentrations of ZEN metabolites were below the sensitivity of the method in this experiment, which prevents an objective evaluation of the rate and direction of changes in ZEN. The above could be attributed to the experimental doses, which did not support the formation of metabolites at detectable concentrations. There is a general absence of published studies whose results could be compared to our findings. Goyarts *et al.* [29] detected α-ZEN in the liver and bile of pigs even at a lower level of exposure to the analyzed mycotoxin, but the experimental animals were intoxicated with both ZEN and DON, at very high DON concentrations in the diet (6680 µg/kg feed). The presence of DON has a significant effect on the absorption and fate of mycotoxins in the gastrointestinal tract [12]. Zearalenone should be converted into a more active metabolite, alpha-zearalenol, and a less active metabolite, beta-zearalenol, by liver subcellular fractions, while evidence of this reaction in other tissues is limited. The available results indicate that in the liver, alpha-zearalenol is a major metabolite in cytosolic fractions, whereas beta-zearalenol is a predominant metabolite in microsome fractions [30]. The rate of the conversion process and the quantities of metabolites produced are determined by numerous factors, such as the age, sex, health status and individual traits of animals, the dose of a toxin or toxins (mixed mycotoxicosis) and the time of exposure. Therefore, the absence or presence of ZEN metabolites in the analyzed tissues may be difficult to explain. More detailed research is needed to determine the optimum conditions for the conversion of ZEN to its detectable metabolites and the effects of the above factors on the process. The highest CF values were noted in the duodenum and the jejunum in the first three weeks of exposure. In Week IV, the highest CF was observed in the jejunum and the descending colon. In the last two weeks of the experiment, CF values were highest in the duodenum and the descending colon. Our results suggest that pre-pubertal gilts accumulate ZEN mainly in the duodenum, jejunum and descending colon.

Mycotoxins (the parent compound and its metabolites) are absorbed, distributed and partially metabolized in the jejunum. The above processes were observed in animals administered plant foods naturally contaminated with mycotoxins. In subjects exposed only to ZEN (at NOAEL doses), the parent compound is not biotransformed or is only insignificantly biotransformed in the digestive tract or intestinal tissues. The presence of α-ZEL and β-ZEL was not determined in toxicological and biochemical analyses. Zearalenone is accumulated in intestinal tissues already in the first week of exposure (Table 1), and this process takes place at a much faster rate than the accumulation of DON [22]. The highest levels of DON were found in the jejunal wall only in the third and fourth week of exposure. The blood plasma concentrations of ZEN and α-ZEL [31] suggest that ZEN levels remained low in the peripheral blood of gilts in the first 28 days of the experiment. Those findings could imply that in the first weeks of exposure, ZEN is transformed to α-ZEL in the bloodstream. In successive weeks, the mycotoxin is distributed to estrogen-sensitive tissues, such as the reproductive system, bone marrow and the central nervous system [32].

According to other studies of pigs, ZEN is degraded by microflora only in the distal section of the large intestine, and transformation processes do not take place in the proximal segment of the intestine [33]. Similar findings were reported by Piotrowska *et al.* [34] in whose study, long-term exposure to doses of ZEN alone exerted an adverse effect on mesophilic aerobic bacteria in the descending colon, but the presence of ZEN metabolites was not reported in the distal section of the large bowel.

The above findings are indirectly confirmed by a study of gilts [35], which demonstrated that ZEN exerts multidirectional effects and inhibits mRNA expression of the gene controlling the constitutional isomer of NOS-1 and the inducible isomer of NOS-2. The above induces specific changes in the gastrointestinal tract of gilts by lowering the concentrations of nitric oxide (NO), which inhibits non-adrenergic and non-cholinergic transmitters [36]. Low levels of NO accelerate esophageal, gastric and intestinal motility, impair gastric accommodation (relaxation, ingestion, accommodation and contraction) and increase tension on the anus, which inhibits stomach emptying and the passage of digesta in the intestines [37], thus slowing down gastric motility [38]. Those changes contribute to antiport activity in the jejunal wall [39] due to slow passage of digesta and prolonged contact between the digesta and intestinal walls. The above can promote the accumulation of ZEN in intestinal walls in the first weeks of exposure, inhibit cell proliferation and slow down apoptosis [40]. The observed changes increased the accumulation of ZEN in intestinal tissues (Table 1) and increased CF values in the first two weeks of exposure, in comparison to other intestinal segments and the liver, excluding the descending colon. Low concentrations of ZEN in feed administered to pre-pubertal gilts slow down motility in the proximal intestine and reduce proliferative activity, which points to a specific defense response to an undesirable substance.

A different scenario is also possible. Our limited knowledge about changes induced by low concentrations of ZEN suggests that exposure to low doses of the mycotoxin can produce unpredictable side effects. This uncertainty results from both dosage and time of exposure. Low doses (NOAEL) can induce surprising responses: (i) undesirable substances, such as mycotoxins, may be “ignored”, and this assumption is similar to the Tregs theory [41], which postulates that Treg cells are not expressed when the number of infectious agents is very low; (ii) the mycotoxin is more likely to be absorbed by the host’s body when it is administered *per os* over long periods of time; and (iii) a compensatory effect is also possible [16] where the activity of undesirable substances is inhibited, and initial functions are restored [12] despite ongoing exposure. Indirect evidence is provided by percentage accumulation of ZEN in different segments of the intestines in successive weeks of exposure. The simultaneous increase in ZEN levels in the duodenum and jejunum was observed only in the first three weeks of the experiment.

The periodic increase in CF values, initially in the duodenum (Weeks I and II) and the jejunum (Weeks I–IV) and then in the duodenum (Weeks V and VI) and the descending colon (Weeks IV–VI) in successive weeks of the experiment (Table 1), prompts further inquiry into whether: (i) the observed fluctuations are influenced by the expression of ERs, which control intestinal function and are distributed in the upper gastrointestinal tract [42], which would validate the effects of steroid substances; and (ii) hyperestrogenism (induced by exposure to ZEN) in pre-pubertal gilts (very low concentrations of 17β-estradiol [43]) induces uncontrolled cell proliferation, inhibits apoptosis and reduces the number of cell adhesion markers in colonic crypts [44]. Female steroid hormones and other estrogenic substances, including ZEN, influence the digestive tract in humans and many animals in various climates. There is much controversy over the significance and distribution of ERs that are implicated in the transport of estrogens in the mammalian gastrointestinal system [45]. Estrogen receptors are unevenly distributed across tissues: (i) ERα are found mainly in the bones, the mammary gland, genitourinary system, cardiovascular system and the central nervous system; (ii) whereas ERβ are distributed mainly in the digestive tract. Estrogen receptors seem to play contrary roles in controlling cell proliferation and differentiation of target tissues. Research has demonstrated that ERβ can modulate ERα expression, inhibit estrogen-dependent proliferation and promote apoptosis [46].

Despite the above, our knowledge about the distribution of ER-positive cells in healthy intestines remains limited [47]. Those cells are most likely to be found in the duodenum, jejunum and the descending colon, which would indicate that CF peaks (Table 1) are correlated with the expression of various ERs. The results of our unpublished study suggest that ZEN ingested with feed can reduce the number and activity of ERα, which, regardless of the above, are sparse in pre-pubertal animals (the ERα:ERβ ratio is estimated at 1:5). 17β-estradiol is an endogenous ligand of ERα [48]; therefore, ERα are unlikely to outnumber ERβ in young gilts where the levels of 17β-estradiol are naturally very low (±6 pg/mL) [42]. ERβ are the predominant receptors in the digestive tract [49], in particular in healthy colonic mucosa [43], and their expression can be somewhat inhibited by ZEN [11]. The cell distribution of ERs and the non-genomic action of estrogenic substances that bind with nuclear receptor ligands are also important [45], and they could explain the decrease in the optical density of ERs due to hyperestrogenism. Zearalenone is a competitive substrate that modulates the activity of enzymes participating in steroidogenesis at the pre-receptor level, whereas α-ZEL is absent or scarce due to slow biotransformation of ZEN.

## 4. Materials and Methods

### 4.1. Experimental Section

All experimental procedures involving animals were carried out in compliance with Polish regulations setting forth the terms and conditions of animal experimentation (Opinion No. 88/2009 of the local Ethics Committee for Animal Experimentation).

### 4.2. Experimental Animals

The experiment was conducted at the Department of Veterinary Prevention and Feed Hygiene, Faculty of Veterinary Medicine, University of Warmia and Mazury in Olsztyn, Poland, on 39 clinically-healthy gilts with initial body weights of 25 ± 2 kg. Gilts were penned in groups with *ad libitum* access to water. The administered feed was tested for the presence of mycotoxins: ZEA, α-ZEL and DON. Mycotoxin levels in the diets were estimated by common separation techniques with the use of immunoaffinity columns (Zearala-Test™ Zearalenone Testing System, G1012, VICAM, Watertown, MA, USA; DON-Test™ DON Testing System, VICAM, Watertown, MA, USA) and high performance liquid chromatography (HPLC) (Hewlett Packard, type 1050 and 1100) with fluorescent and/or UV detection techniques. The limit of detection was 2 µg/kg for ZEN.

### 4.3. Experimental Design

The animals were divided into an experimental group (E, *n* = 18) and a control group (C, *n* = 21). Group E animals were orally administered ZEN at 40 μg/kg BW. Group C pigs were fed a placebo. The animals were fed complete diets in a restricted feeding regimen (Table 1). The daily dose of ZEN (40 µg/kg BW) was administered to each animal individually, which corresponds to exposure to 957–1014 µg ZEN/kg of a complete diet, depending on daily feed intake. Zearalenone was biosynthesized, purified and standardized by the Department of Chemistry of the Poznań University of Life Sciences. The experiment covered a period of 42 days. The mycotoxin was administered *per os* daily, in gelatine capsules before the morning feeding. The ZEN dose was adjusted to the body weight of the experimental animals. The mycotoxin was administered in capsules to prevent problems associated with uneven feed intake. Mycotoxin samples were diluted in 300 μL of 96% ethyl alcohol (96% ethyl alcohol, SWW 2442-90, Polskie Odczynniki Chemiczne SA, Gliwice, Poland) to the required dose level (based on body weight). Final solutions were stored at room temperature for 12 h to evaporate the solvent. The animals were weighed every 7 days to update mycotoxin doses for each gilt. Three animals from the experimental group and the control group each were sacrificed on Days 1, 7, 14, 21, 28, 35 and 42 (a total of 6 gilts in each week of exposure), excluding day 1, when only 3 control group animals were scarified. Every week, tissue samples were collected from the porcine gastrointestinal tract (duodenum, first and middle sections, jejunum, ileum, cecum, ascending colon, transverse colon, descending colon, middle section, and the liver, left lobe). Tissue samples were collected from the whole intestinal cross-sections.

### 4.4. Chemicals

A chromatographic analysis of ZEN, α-ZEL and β-ZEL was performed by the Department of Chemistry, Poznań University of Life Sciences, Poland. Standard and organic solvents (HPLC grade) for analyses of ZEN, α-ZEL and β-ZEL were supplied by Sigma-Aldrich (Steinheim, Germany). All chemicals for the extraction and purification of mycotoxins were purchased from POCh (Gliwice, Poland). Water for the HPLC mobile phase was purified using the Milli-Q system (Millipore, Bedford, MA, USA).

### 4.5. Tissue Samples

Post-mortem tissue samples were collected from the duodenum, jejunum, ileum, cecum, ascending colon, transverse colon, descending colon and the liver after rinsing with phosphate buffer. Pure tissues were stored at −80 °C until shredding and extraction.

### 4.6. Extraction and Purification

Tissue samples (2 g) were homogenized with 9 cm^3^ of methanol for 4 min; they were transferred to centrifuge tubes with a minimal amount of the sediment, and the remainder was combined with 5 cm^3^ of methanol and repeatedly homogenized. The extracts were combined and centrifuged for 10 min. The supernatant was transferred to a graduated test tube; its volume was read from the scale, and it was evaporated under a stream of nitrogen at a temperature of 60 °C to 0.1–0.2 cm^3^ of water. The evaporated product was combined with 2 cm^3^ of deionized water, 2 cm^3^ of 0.2 M sodium acetate and 500 µL of 3-glucuronidase solution. After 16 h of incubation at 37 °C, the reaction was stopped by adding 0.5 cm^3^ of acetonitrile. The mixture was quantitatively transferred to a centrifuge tube; it was combined with 10 cm^3^ of deionized water and centrifuged for 10 min at 3500 rpm. The supernatant was applied to the Zearala-Test™ column at the rate of 1–2 drops per second. Mycotoxins were eluted from the column with 1.5 mL of methanol; the solvent was evaporated, and the residue was reconstituted with 1 mL of methanol for HPLC analysis.

### 4.7. HPLC Analysis of Zearalenone and Its Derivatives

The concentrations of ZEN and its derivatives were determined by HPLC with the use of the Waters 2695 Separations Module, Waters 2475 Multi λ Fluorescence Detector and Waters 2996 Array Detector. Excitation and emission wavelengths were 274 and 440 nm, respectively. The reverse-phase column was C-18 Nova Pak (3.9 × 150 mm), and the mobile phase was acetonitrile-water-methanol (46:46:8, *v/v/v*) at a flow rate of 0.5 mL·min^−1^. The mycotoxins were quantified by measuring the retention time of the peaks based on the relevant calibration curves (correlation coefficient of 0.9996 for ZEN, 0.9989 for α-ZEL and 0.9976 for β-ZEL). Detection limits were determined at 0.001 µg·g^−1^ for ZEN, 0.003 µg·g^−1^ for α-ZEL and 0.002 µg·g^−1^ for β-ZEL.

### 4.8. Carryover Factor

The daily dose of ZEN (40 µg/kg BW) was administered to each animal individually, which corresponds to exposure to 957–1014 µg ZEN/kg of a complete diet, depending on daily feed intake.

Mycotoxin concentrations in tissues were given in terms of the dry matter content of the samples. The carryover factor was calculated as follows:

Carryover factor=toxin concentration in tissue [ngg]toxin concentration in diet [ngg]

### 4.9. Statistical Analysis

Significant differences in ZEN concentrations between tissue samples were evaluated by Tukey’s test. The results were presented as medians ($\bar{x}$) ± SD. Data were processed in the Statistical Package for the Social Sciences.

## 5. Conclusions

The following conclusions can be formulated based on the results of this study: (i) *per os* exposure to subclinical doses of ZEN does not induce detectable biotransformation processes; (ii) changes in CF values in different intestinal segments during exposure to subclinical doses of ZEN in different weeks of the study could be determined by the location and expression of ERβ; (iii) ZEN is accumulated in the jejunum and the descending colon in different weeks of exposure, and it is accumulated in the duodenum throughout the entire period of exposure.

## Abbreviations

ZEN	zearalenone
α-ZEL	α-zearalenol
β-ZEL	β-zearalenol
CF	carryover factor
EDCs	endocrine disrupting chemicals
ERs	estrogen receptors
VBS	values below the sensitivity of the method
BW	body weight
DON	deoxynivalenol
SD	standard deviation
NOAEL	no observable adverse effect level
PPB	parts per billion
